# Uncovering the Evolution of Low-Energy Plasmons in Nanopatterned Aluminum Plasmonics on Graphene

**DOI:** 10.1021/acs.nanolett.2c01512

**Published:** 2022-07-12

**Authors:** Kenan Elibol, Peter A. van Aken

**Affiliations:** Stuttgart Center for Electron Microscopy, Max Planck Institute for Solid State Research, Heisenbergstr. 1, 70569 Stuttgart, Germany

**Keywords:** charge-transfer plasmon, localized surface plasmons, electron energy-loss spectroscopy, nanofabrication, graphene, scanning transmission electron microscopy

## Abstract

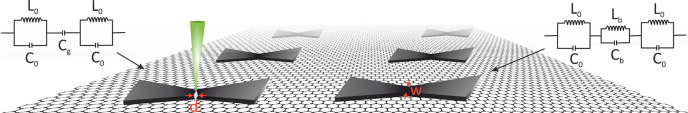

We report adjusting the charge-transfer-plasmon (CTP) resonances of aluminum (Al) bowties on suspended monolayer graphene via controlled nanofabrication and focused electron-beam irradiation. CTP resonances of bowties with a conductive junction blue-shift with an increase in junction width, whereas their 3λ/2 and λ resonances barely red-shift. These plasmon modes are derived and confirmed by an LC circuit model and electromagnetic simulations performed with boundary-element and frequency-domain methods. A monotonic decay of the CTP lifetime is observed, while the junction width is extended. Instead, the lifetimes of 3λ/2 and λ resonances are nearly independent of junction width. When the junction is shrunk by electron-beam irradiation, all antenna resonances red-shift. Having created an electron-beam-induced sub 5 nm gap in bowties, we monitor the unambiguous transition of a CTP into a bonding-type gap mode, which is highly sensitive to the separation distance. Meanwhile, the 3λ/2 and λ resonances evolve into dipolar bright and dipolar dark modes.

When a conductive path is formed between two metallic nanoparticles, a charge-transfer plasmon (CTP) emerges.^1−6^ The electron transport via quantum tunneling between nearly touching nanoparticles also enables excitation of the CTP at optical frequencies.^6−9^ CTPs are of significant importance for applications, particularly in single-molecule sensing and functional devices such as ultrafast nanoswitches and terahertz-frequency photonic devices.^8,10,11^ Despite its exciting features and applications, the lack of a spectrally tunable CTP hinders its further use in device applications.^12^

Nanofabrication through electron-beam lithography (EBL) provides a rational route for tuning CTP resonances in nanopatterned plasmonic devices.^1,13−16^ However, it is not enough to explore the evaluation of CTPs and other higher-order antenna modes into different plasmon resonances, while the junction width of antennas is altered. As a complementary method, a focused electron beam has been employed to manipulate the junction width of gold bowtie antennas on SiN_*x*_ membranes.^14^ In contrast to gold and silver, which have resonances in the visible and near-infrared regions, aluminum (Al) is an earth-abundant and low-cost plasmonic material that finds applications in visible and ultraviolet (UV) regions of the spectrum due to its higher plasma frequency.^17−20^ Although a natural oxide forming on the surface of Al nanostructures induces a thickness inhomogeneity and a red shift in their LSPRs, it acts as a protection layer for the metallic Al and enables fabricating robust plasmonic devices.^17,21^ This thin natural oxide layer (3–5 nm)^22^ also limits the creation of coupled Al antennas with smaller gap sizes of <5 nm. Consequently, CTP resonances and their evolution have not been studied for nanopatterned Al plasmonics with a sub 5 nm gap opening.

Electron energy-loss spectroscopy (EELS) with a monochromated scanning transmission electron microscope (STEM) is a powerful method for mapping low-energy plasmon excitations such as CTPs with a high spectral resolution.^23−26^ Most of the EELS studies on CTP resonances have been performed using nanopatterned plasmonic devices fabricated on top of a 30 nm thick SiN_*x*_ membrane, leading to a red shift and increased line width of the CTP.^13−15,27^ Although graphene is found to be an excellent substrate for plasmonic nanoparticles,^28^ the CTP resonances of nanopatterned plasmonic devices fabricated on atomically thin suspended membranes, such as monolayer graphene and hexagonal boron nitride (h-BN), have not been studied to date. It has been found that a conductive substrate, such as a gold film, can induce the excitation of CTPs in nanoparticle dimers with a separation distance of 15 nm,^16^ but there has not been a report demonstrating the excitation of CTPs in nanoparticle dimers located on bare suspended graphene.^29^ On the other hand, a gate-controlled monolayer graphene on a solid support, which enables tuning the density of carriers by applying bias through the gate, has been used to enhance the tunability of CTPs.^29−32^

To address the shortcomings in revealing low-energy excitations of Al plasmonics with sub 5 nm gaps, we fabricated for the first time Al antenna arrays including bowtie structures with varying bridge widths onto suspended monolayer graphene membranes via EBL and then manipulated their conductive junctions with a focused electron beam. The evolution of plasmon modes in Al bowties with small gaps has been monitored by low-loss EELS measurements with a monochromated STEM. Our findings ascertain that the CTP resonances of bowties with a conductive junction depend strongly on the kinetic inductance of the junction between the nanoprisms. CTP resonances blue-shift, while their lifetimes, which are in the femtosecond range, decay monotonically with an increase in junction width. Unlike the case for CTPs, the 3λ/2 and λ resonances slightly red-shift. When the junction width is reduced by electron-beam irradiation, CTPs and other higher-order antenna modes explicitly red-shift. With the complete removal of the junction by a focused electron beam, the CTP disappears and a new bonding-type gap mode emerges. Additionally, the 3λ/2 and λ resonances are transformed into dipolar bright and dipolar dark localized surface plasmon resonances (LSPRs). All of these plasmon modes are analyzed and confirmed by means of an LC circuit model and full electromagnetic simulations based on the boundary-element method (BEM) and finite-difference time domain (FDTD).

Figure 1a shows a schematic of antenna arrays on suspended graphene. As depicted, a focused electron beam is used to open a nanoscale gap in Al bowties. Here, we model each structure as a simple LC tank oscillator circuit to explain the plasmon resonances measured by EELS. The details of the LC circuit model reported earlier by Duan et al.^15^ are given in Supplementary Methods (see also Figure S1). A coupled-inductor/capacitor model is considered to clarify the plasmon modes of bowties with a conductive junction, while the bowties with a gap are modeled as a coupled capacitor. The dimensions of antenna arrays including 30 nm thick bowties on CVD-grown monolayer graphene are adjusted by EBL (see Supplementary Methods and Figures S2–S4). A bowtie with a junction width of 27.4 nm and its corresponding model are shown in Figure 1b,c. The junction width of the bowtie measured from the volume plasmon map is smaller than the width measured directly from the HAADF image (see Figure 1d). This is due to the oxide layer covering the metallic aluminum (see Figure S5). Figure 1e shows the EEL spectra acquired at different positions of the bowtie. The red, blue, and black arrows point to the most pronounced plasmon excitations of the bowtie. The green arrow shows the interband transition (IBT) of Al at ∼1.5 eV.^18^Figure 1c indicates a model corresponding to the experimental structure, and the red curves in Figure 1e shows simulated EEL spectra derived from different positions on the model. Quantitatively, the plasmon resonances in both experimental and simulated EEL spectra coincide (Figure 1e). Although the experimental and simulated EELS maps acquired at different energies have similar patterns (see Figure 1f,g), they do not provide enough information on the type of modes observed. Instead, the eigenmode analysis implicitly allows discerning different plasmon resonances (see Figure 1h). The peak marked by a red arrow in Figure 1e is thus found to be a CTP that lies at low energies. The CTP, which stems from the charge flow from one prism to the other, is also classified as a λ/2 resonance, when antenna theory is adopted for the classification of longitudinal modes. The computed eigenmodes show that the plasmon resonances excited at 2.27 and 2.44 eV correspond to a 3λ/2 and a dark λ resonance, respectively (see Figure 1g,h).

**Figure 1:**
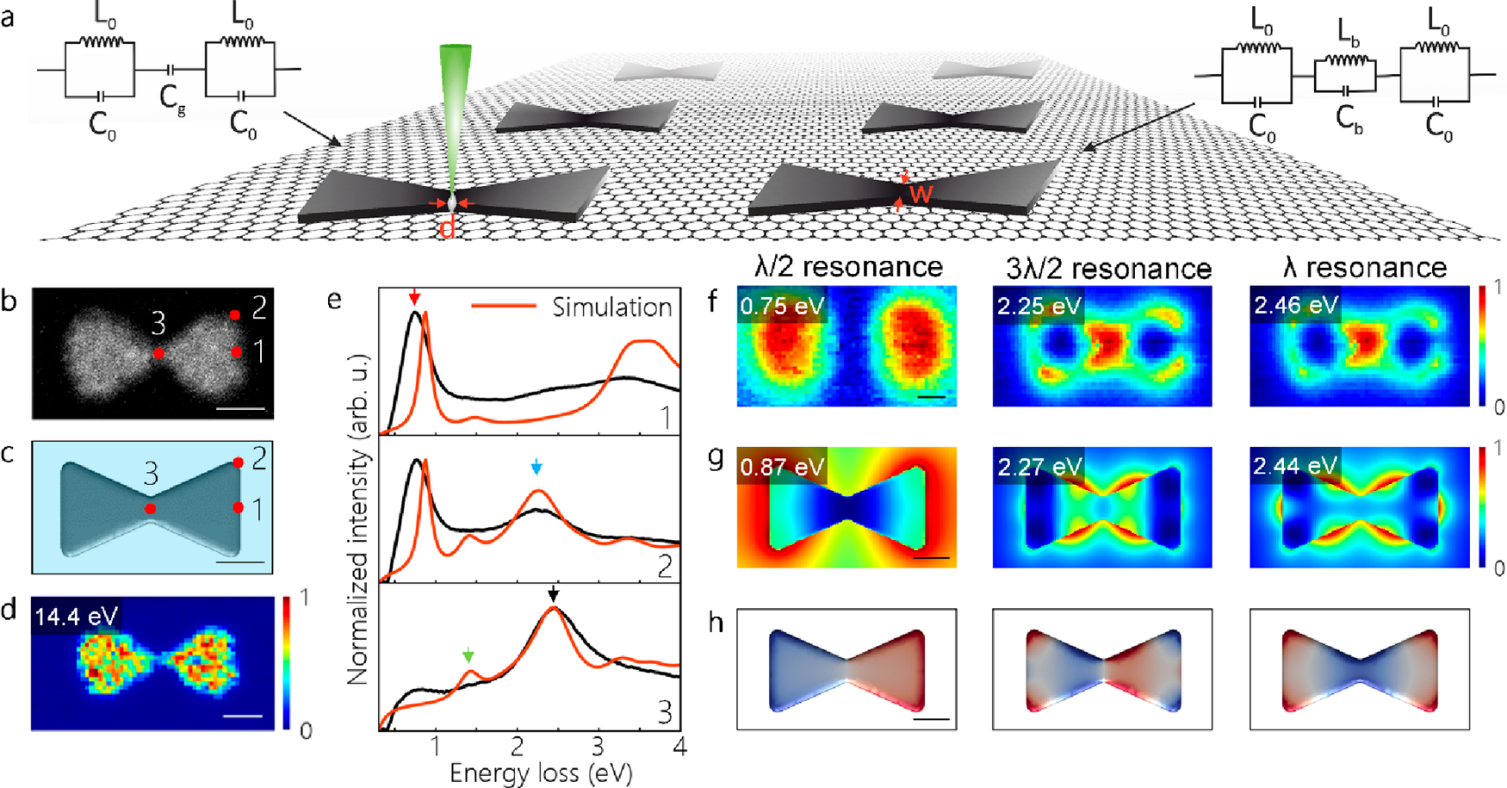
(a) Schematic of nanopatterned Al structures on suspended graphene and LC circuit models corresponding to the structures with and without a gap. Here, the gap is created by a focused electron beam. (b, c) HAADF image of a bowtie antenna with a conductive junction and its corresponding model used in BEM simulations, respectively. The effective medium surrounding the bowtie is shown in blue in (c). (d) Volume plasmon map for the bowtie in (b). (e) Experimental (black curves) and simulated (red curves) EEL spectra obtained at the positions marked with red dots in (b) and (c). The red, blue, and black arrows mark the λ/2 (CTP), 3λ/2, and λ resonances, while green arrows show the IBT. (f, g) Experimental and simulated EELS maps. (h) Computed eigenmodes corresponding to the CTP, 3λ/2, and λ resonances, respectively. The scale bars are 50 nm.

We assess the experimental and simulated plasmon resonances described in Figure 1 as a function of junction width (see Figure 2a,b). The width of junctions in the experimental structures are changed from 13.7 to 62.6 nm by varying the beam dose from 1200 to 2964 μC/cm^2^ during the EBL process. While the 3λ/2 and λ resonances do not exhibit a drastic change with the increase of junction width, the λ/2 resonance blue-shifts due to the reduction of the kinetic inductance in the junction (see Figure 2a). The blue shift of the λ/2 resonance is further confirmed by BEM simulations of EEL spectra and the LC circuit model fitted to the experimental and simulated data (see Figure 2a,b). Unlike the λ/2 resonant mode, the LC circuit model shows a constant energy for the λ resonance. This is because the LC tank oscillators (Al nanoprisms) are independent of junction width. Thus, the resonance energy is predicted by *E* = *ℏ*ω_0_ = *ℏ/*, where *ℏ* is Planck’s constant, *L*_0_ is a nanoinductor, and *C*_0_ is a nanocapacitor. In fact, there should be a slight change in *L*_0_ and *C*_0_, because of the change in junction width as well as the redistribution of the charges and fields. Although the LC circuit model does not determine the details stemming from these variations, BEM simulations indicate a slight blue shift in the λ resonance of bowties with greater junction widths. The 3λ/2 resonance is clarified remarkably well by the LC model, as both BEM simulations and the LC circuit model demonstrate a slight blue shift of the 3λ/2 resonance. Figure 2c,d shows the experimental and simulated lifetimes for all antenna resonances as a function of junction width. The lifetimes of these resonances are calculated using the Heisenberg uncertainty relation^33^ τ = *ℏ*/fwhm, where fwhm is the line width obtained from fitting (see Figure S6). Here, the CTP lifetime reduces consequentially to ultrafast time scales with an increase in the junction width, while the lifetimes of 3λ/2 and λ resonances are almost independent of the junction width. This implies that the kinetic inductance of the junction is also critical for tuning the lifetime of the CTP. The influence of junction width on λ/2, 3λ/2 and λ resonances is demonstrated further in the simulated EEL spectra (see Figure S7). In BEM simulations, a graphene membrane is not involved. Instead, we apply an effective medium approach for the simplicity of complex simulations. To find out the effect of graphene on plasmon modes of Al bowties, we perform additional electromagnetic simulations based on FDTD (Figure S8). FDTD simulations show that monolayer graphene induces a red shift of 0.09 eV in the CTP resonance of an Al bowtie with a conductive junction.

**Figure 2:**
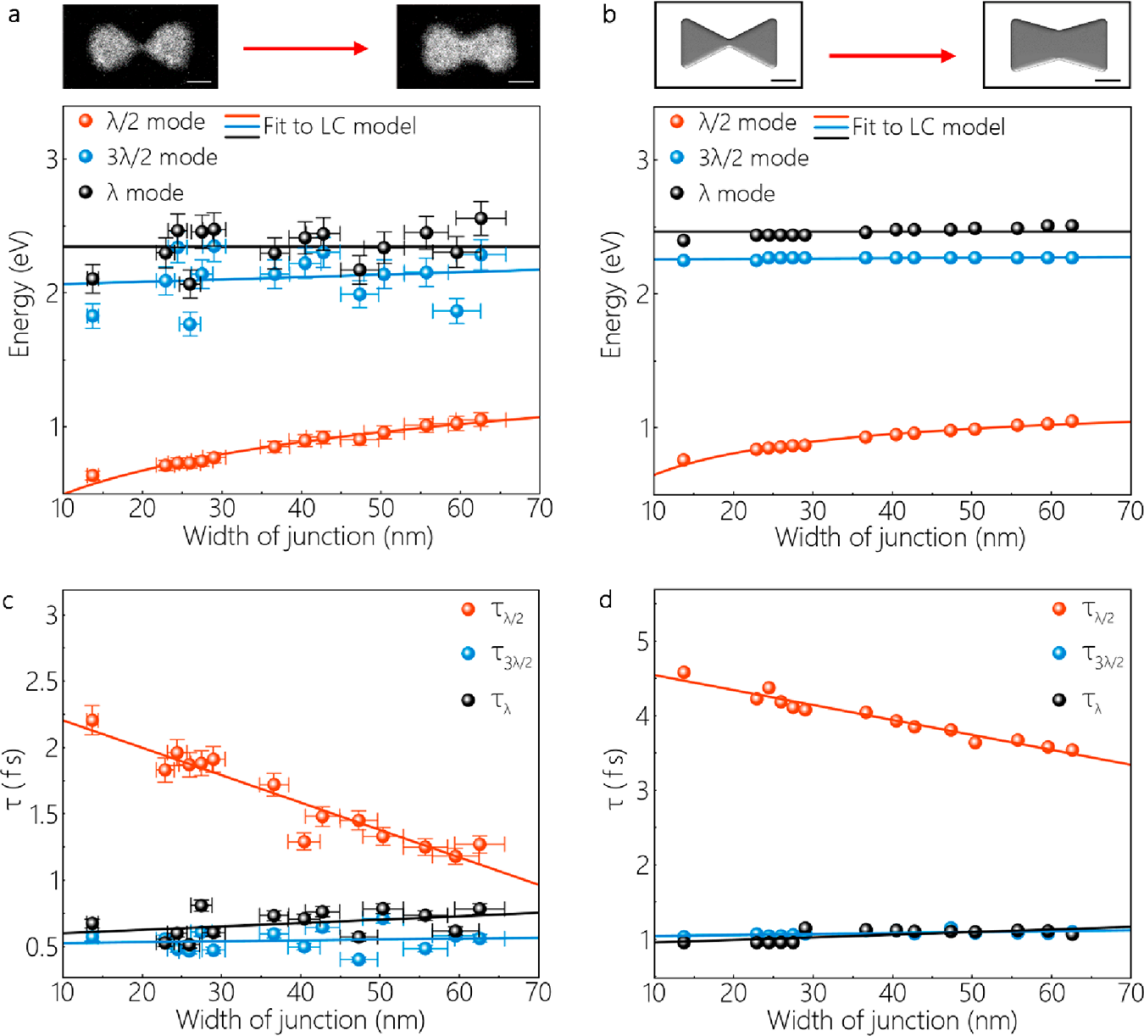
(a, b) Experimental and simulated λ/2, 3λ/2, and λ resonances as a function of junction width, respectively. HAADF images and their corresponding models are shown above these plots. The scale bars are 50 nm. (c, d) Experimental and simulated lifetimes of λ/2, 3λ/2, and λ resonances as a function of junction width, respectively.

To ascertain further the effect of the junction width on the CTP resonance, we measure plasmon resonances of Al bowties manipulated with a focused electron beam accelerated to 200 keV kinetic energy (see details in Supplementary Methods). A bowtie with a junction width of 40.5 nm and its corresponding model are displayed in Figure 3a,b. Here, the electron probe is placed at various locations on the junction area to ensure beam damage of the bowtie junction. The knock-on damage induced by the focused electron beam establishes a partially open gap at the junction area and thus reduces the junction width from 40.5 to 24.5 nm (see Figure 3c–f). Notably, shrinking the junction width results in a clear red shift of λ/2, 3λ/2, and λ resonant modes due to the increase in kinetic inductance (see Figure 3g). The red shifts in 3λ/2 and λ resonances are larger in the experimental data due to the deviations in the dimensions of the experimental structure and the dielectric function of Al used in BEM simulations (see Figure 3h). Similarly to the bowtie in Figure 1b, EELS maps and eigenmodes in Figure 3i–k confirm that the bowtie with a 40.5 nm junction width has λ/2, 3λ/2, and λ resonant modes. Here, the EELS maps of the manipulated bowtie show almost the same plasmon resonances observed in the bowtie before electron-beam manipulation (see Figure 3l–n). Interestingly, the eigenmode for the plasmon resonance at 0.84 eV displays a mixed mode consisting of a λ/2 resonance and a bonding-type gap mode,^34^ which is visible at the area including a gap (see Figure 3n). This is the first signature demonstrating a transition of the λ/2 resonance into a bonding-type gap mode. Moreover, the eigenmode computed for the 3λ/2 resonance suggests a transition from the 3λ/2 resonance to a dipolar bright mode. In contrast to the λ/2 and 3λ/2 resonances, there is no distinct difference in the λ resonance of the bowtie before and after electron-beam manipulation (see Figure 3k,n).

**Figure 3:**
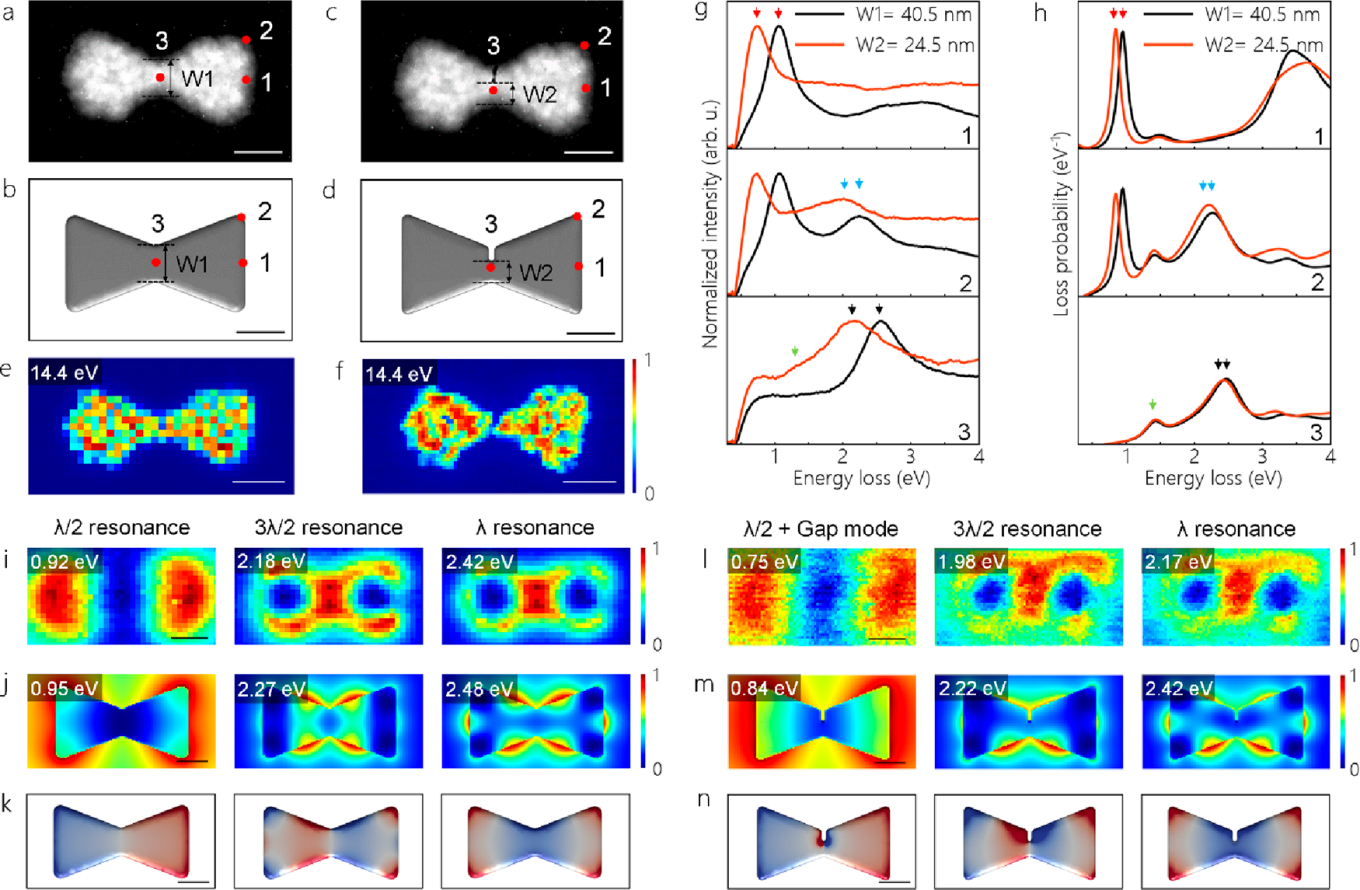
(a, b) HAADF image of a bowtie with a junction width of 40.5 nm and its corresponding model used in BEM simulations. (c, d) HAADF images of the same bowtie, when its junction width is diminished to 24.5 nm. The corresponding model is used in BEM simulations. (e, f) Volume plasmon maps for bowties in (a) and (c). (g, h) Experimental and simulated EEL spectra extracted at positions marked with red dots in (a)–(d), respectively. The red, blue, and black arrows point to the λ/2, 3λ/2, and λ resonances, while green arrows show IBTs. (i, j) Experimental and simulated EELS maps for the bowtie in (a). (k) Computed eigenmodes corresponding to the λ/2, 3λ/2, and λ resonances, respectively. (l, m) Experimental and simulated EELS maps for the bowtie in (c). (n) Computed eigenmodes corresponding to the λ/2 + gap mode, 3λ/2, and λ resonances, respectively. The scale bars are 50 nm.

We now move on to the plasmon excitations of a bowtie after it has been divided into two nanoprisms by a focused electron beam (see Figure 4a–d). Figure 4g–k shows detailed analyses of λ/2, 3λ/2, and λ resonances of a bowtie without a gap. The volume plasmon maps and line profiles recorded along the white dashed lines on HAADF images confirm that the nanoprisms are completely separated by the electron-beam irradiation (see Figure 4e,f and Figure S9). The average established gap size is ∼3.4 nm (see Figure S10). The variations in the separation distance are due to a sample drift and multiple irradiations of the same locations. The surface plasmon resonances for gold and silver are observed at energies lower than the IBT threshold. In contrast to these materials, the surface plasmon resonances in Al can be excited at energies lower or higher than the IBT energy.^18^ In the previous electron-beam-manipulated bowtie structure including both a conductive junction and a partial nanogap (see Figure 3), we have observed a mixed mode including a λ/2 resonance and a gap mode at 0.84 eV, which is lower than the IBT energy of Al (see Figure 3). We now trigger the switching of λ/2, 3λ/2, and λ resonances through the entire uncoupling of nanoprisms with a focused electron beam (see Figure 4g,h). By establishment of a gap of ∼3.4 nm in the bowtie, the spectrally steep λ/2 resonance vanishes and two new modes occur at energies close to the 3λ/2 and λ resonances (see Figure 4g–n). These new plasmon resonances of the electron-beam-manipulated bowtie are corroborated by revealing its EELS maps and computed eigenmodes (see Figure 4l–n). The EELS maps acquired at 2.16 and 2.25 eV (see Figure 4l,m) are similar to the EELS maps of 3λ/2 and λ resonances (see Figure 4i,j). Conversely, the plasmon mode visualized at 1.24 eV is implicitly different from the λ/2 resonance of the initial bowtie structure (see Figures 4i,l). As shown in both experimental and simulated EELS maps created at 1.24 and 1.23 eV, respectively, there is an enhancement of the intensity in the area where the nanogap is formed. The additional field enhancement is due to a strong localization of surface charges within the gap. The increase of surface charges at the hot spot is attributed to the hybridization of the bonding dipole and hexapole modes in closely spaced nanoprisms.^35^ The eigenmode of the plasmon resonance at 1.23 eV shows the emergence of a bonding-type dipole gap mode, when a gap of <5 nm is formed (see Figure 4n). Since the suspended graphene monolayers used in this work are not gated, they are charge-neutral on average despite the charge carriers, which are locally available.^36,37^ Hence, the CTP cannot be excited in these disconnected bowties on suspended graphene. The modes appearing at 2.25 and 2.42 eV are dipolar bright and dipolar dark LSPRs (see Figure 4n). The plasmon modes (3λ/2, λ, dipolar bright, and dipolar dark) observed in connected and disconnected nanoprims are in good agreement with previous literature.^15,38,39^ The discrepancy between the experimental and simulated EELS maps around energies of ∼2.2 and ∼2.5 eV is due to a mismatch between the experimental structure and the model used in the simulations. Unlike the model with perfect edges and a homogeneous thickness distribution, the edges of experimental structures are slightly inclined and the junction area in connected nanoprisms is thinner than the center of these nanoprisms (see Figure S4). To clarify this, we compare the simulated EELS maps of 20 and 10 nm thick bowties with and without a junction (see Figures S11 and S12). Similarly to the experiment, we observe a strong enhancement of the intensity around the junction and the gap in the simulated EELS maps of 10 nm thick structures. This finding demonstrates that the thinner junction area and the inclined edges of the experimental structures result in an enhanced intensity around the junction and the gap in EELS maps. Since BEM simulations do not involve the graphene membrane, we perform FDTD simulations for a bowtie with a 3.4 nm gap on graphene to figure out the influence of the graphene membrane. The simulated surface-charge distribution and electric-field map also verify that this is a dipole gap mode, which is strongly suppressed for gap distances larger than 5 nm (Figures S13 and S14). Unlike dipolar LSPR modes, the gap mode, which is sensitive to the separation distance, is only excited when a gap of <5 nm is formed between two Al nanoprisms (see Figure 4l–n and Figure S14). To the best of our knowledge, we demonstrate here for the first time that a gradual transition of antenna modes into a gap mode and dipolar LSPR modes becomes possible upon creating a sub 5 nm gap in the junction area of Al bowties.

**Figure 4:**
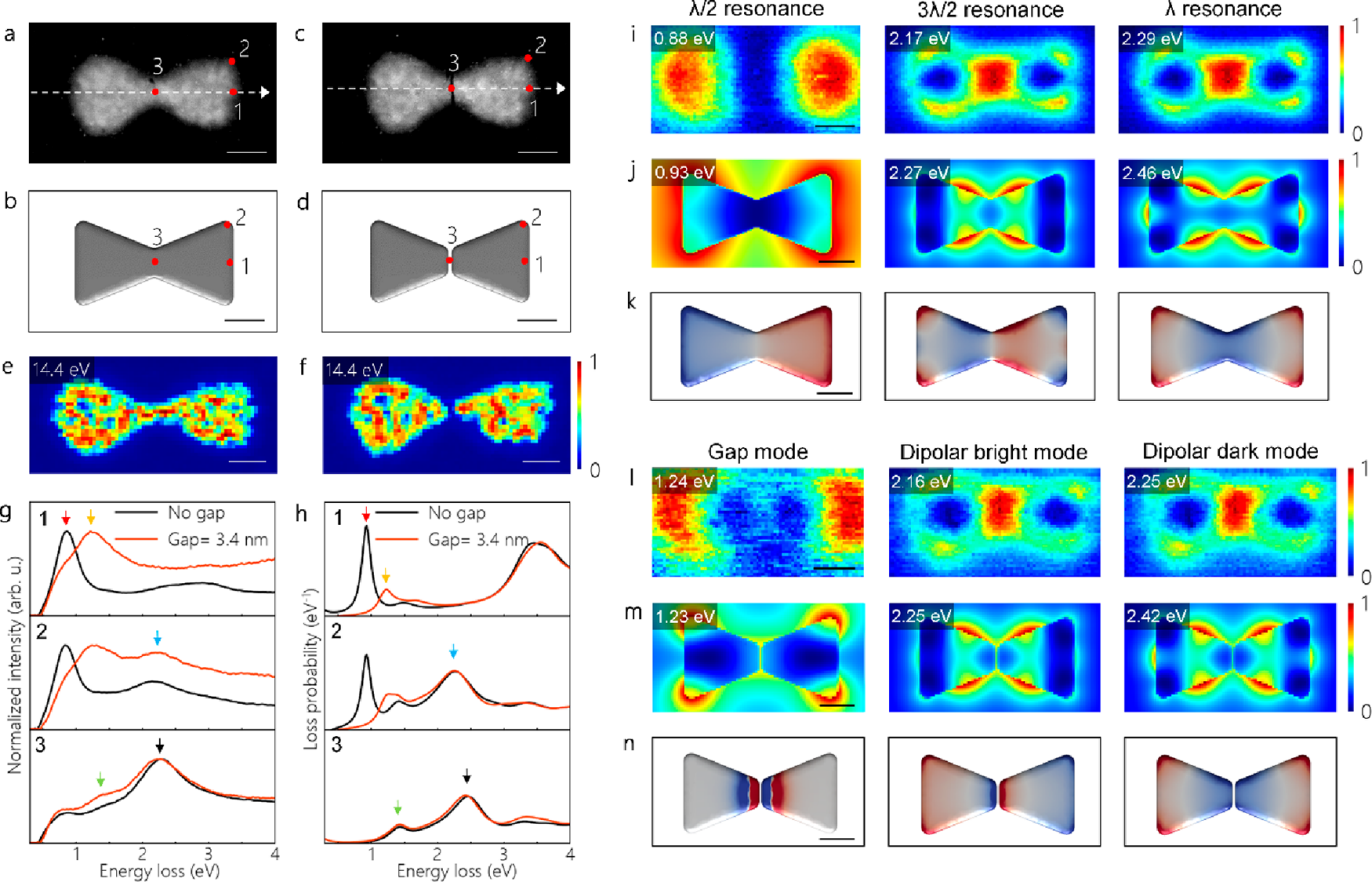
(a, b) HAADF image of a bowtie with a junction width of 36.6 nm and its corresponding model used in BEM simulations. (c, d) HAADF image of the same bowtie after creating a 3.4 nm gap and its corresponding model used in BEM simulations. (e, f) Volume plasmon maps for the bowties in (a) and (c). (g, h) Experimental and simulated EEL spectra extracted at the positions marked with red dots in (a)–(d), respectively. The red and orange arrows point to the CTP and gap modes, respectively. The blue arrows point to the 3λ/2 and dipolar bright LSPR modes, while the black arrows point to the λ and dipolar dark LSPR modes. The green arrows show the IBT. (i, j) Experimental and simulated EELS maps for the bowtie in (a). (k) Computed eigenmodes corresponding to the λ/2, 3λ/2, and λ resonances, respectively. (l, m) Experimental and simulated EELS maps for the bowtie in (c). (n) Computed eigenmodes corresponding to the gap mode, dipolar bright mode, and dipolar dark mode, respectively. The scale bars are 50 nm.

To clarify the findings reported in Figures 3 and 4, we have gradually manipulated the junction of an Al bowtie via electron-beam irradiation (see Figure S15a–i). Here, we first reduce the junction width and then remove the junction completely. Similarly to the results shown in Figure 3, λ/2, 3λ/2, and λ resonances of the bowtie with a shortened junction width red-shift (see Figure S15b,e,j,k). When a gap of ∼3.4 nm is formed between two nanoprisms (Figures S16 and S17), λ/2, 3λ/2, and λ resonances switch to a low-energy gap mode, dipolar bright LSPR, and dipolar dark LSPR (see Figure S15, S18, and S19).

In summary, we tuned plasmon modes, particularly the CTP, of Al bowties on suspended graphene by controlled nanofabrication and electron-beam irradiation. An increase in the junction width led to a blue shift of the CTP energy, while it concomitantly induced a slight red shift in the 3λ/2 and λ resonances. The observed plasmon resonances were described in detail via an LC circuit model and electromagnetic simulations based on BEM and FDTD. The CTP lifetime, which is in the femtosecond range, is lower for bowties with larger junction widths, where BEM simulations further confirmed the monotonic decrease of the CTP lifetime. Electron-beam manipulation of junctions in bowties enables adjusting CTP resonances and reveals the evaluation of their antenna modes into different plasmon resonances. By reduction of the junction width via a focused electron beam, an explicit red shift is observed of the CTP, 3λ/2, and λ resonances of bowties. When the conductive junction between two nanoprisms is removed completely by electron-beam irradiation, the CTP disappears and a low-energy bonding-type gap mode emerges at slightly higher energy, which is lower in energy than the interband transition energy of Al. The gap mode exhibits a strong dependence on the separation distance and appears only when the distance is smaller than 5 nm. In contrast, the 3λ/2 and λ resonances turn into dipolar bright and dipolar dark LSPR modes at energies where 3λ/2 and λ resonances are excited. Consequently, both approaches facilitate active control of CTP resonances of Al plasmonics on the femtosecond time scale, which is crucial for applications, especially for ultrafast nanoswitches.

## Supporting Information Available

Methods (sample fabrication, electron-beam manipulation of bowties, HRTEM, STEM, and EELS measurements and electromagnetic simulations) and additional figures as described in the text (PDF)
