# Supplementary material for: Uncovering the Evolution of Low-Energy Plasmons in Nanopatterned Aluminum Plasmonics on Graphene
Source: Nano Lett. 2022 Jul 12;22(14):5825–31. doi: 10.1021/acs.nanolett.2c01512 (PMC9335878; doi:10.1021/acs.nanolett.2c01512)
Supplement: Supplementary file 1 — nl2c01512_si_001.pdf [file nl2c01512_si_001.pdf]

Supporting Information:

Uncovering the evolution of low-energy plasmons in nanopatterned aluminum plasmonics on graphene

Kenan Elibol and Peter A. van Aken*

Stuttgart Center for Electron Microscopy, Max Planck Institute for Solid State Research Heisenbergstr. 1, 70569 Stuttgart, Germany.

*Corresponding author: k.elibol@fkf.mpg.de

SUPPLEMENTARY METHODS

Sample preparation. The arrays of Al bowties were fabricated on CVD-grown monolayer graphene on a Cu foil (Graphenea Inc.) by EBL (see Supplementary Figure S1). Firstly, the CVD graphene grown on Cu foil (~4 mm×4 mm) was cleaned with acetone and then rinsed in isopropanol. The CVD graphene on Cu was then placed on a flat Si substrate and coated with an 80–90 nm-thick PMMA (2% PMMA 950k in anisole) by a spinner running at 6000 rpm (acceleration rate of 2000 rpm/s) for 35 s. After the spin coating of the resist, the samples located

on a hot plate were heated at 160 °C for 4 min. The resist was patterned by a Raith eLine EBL system with a 7.5 μm objective aperture and an acceleration voltage of 15 kV. The working distance, the beam current and the areal dose were 9 mm, 19.4 pA and 1200 $\mu C/cm^2$, respectively. The exposed resist was developed in methyl isobutyl ketone (MIBK)/isopropyl alcohol (IPA) solution (3:1) at 0 ºC for 30 s. Following the development, the exposed resist was cooled by a custom-designed cooler enabling chilling in a nitrogen environment and dried by a nitrogen spray gun. Subsequently, a 30 nm-thick aluminum (99.99% purity) layer was deposited on the sample by a thermal evaporator (Univex 1) with a base pressure of $\sim 1.6\times10^{-6}$ mbar. The deposition rate of Al was measured to be ~2 Å/s on a quartz crystal microbalance. To lift-off the metal on unexposed PMMA, the sample was immersed in N-Methyl-2-pyrrolidone (NMP) heated at 60 °C for ~30 min and rinsed with acetone and isopropanol, respectively. Finally, the sample was dried by a nitrogen spray gun. The carbon support films with holes (quantifoil) attached to the Au TEM grids were covered with a 10 nm-thick Pt film by a sputter coater (Leica), before the transfer of Al bowties fabricated on a graphene/Cu stacks. The TEM grids on the sampled were positioned by a home-designed micromanipulator. The adhesion between the quantifoil and graphene was provided with a drop of IPA. The Cu foil was etched away by a 10% ammonium persulfate (APS) solution applied for ~3 hours. The TEM grids carrying the nanocavity/graphene stacks were then rinsed with IPA. SEM images of the samples were recorded by a Zeiss Gemini DSM 982 with a cold field-emission gun and an In-lens detector at an acceleration voltage of 5 kV. The working distance was 14 mm in the measurements.

Electron-beam manipulation of bowties. A JEOL ARM200F STEM/TEM with a probe Cs-corrector was used for manipulating the junctions with a focused electron beam. To induce knock-on damage on Al bowties, a focused electron beam accelerated at 200 kV was placed on the sample

for ~1 min. We created nanoscale gaps by placing the electron beam at different locations on the bowtie structures. Some points were irradiated multiple times for a complete separation of the nanoprisms. The probe size was set to 2C corresponding to the beam diameter of 0.27 nm to enhance the beam current. The beam current and current density were 15 μA and 83 pA/cm^2 during the experiments.

HRTEM, STEM and EELS measurements. HRTEM and STEM measurements were performed by a JEOL ARM200F TEM with a cold field-emission gun and a post-specimen spherical aberration corrector (C_s) at under-focus conditions. All HRTEM images were acquired at the acceleration voltage of 80 kV. The sub-electron-volt-sub-angstrom microscope (Zeiss SESAM) equipped with a Schottky field-emission gun, an electrostatic OMEGA-type monochromator, a high-dispersion and high-transmissivity MANDOLINE filter was used for low-loss EELS measurements. All the measurements were performed at an acceleration voltage of 200 kV. The energy resolution, energy dispersion and EELS collection semi-angle were 0.17 eV, 0.015 eV/px, and 0.7 mrad, respectively. The pixel dwell time was set to 0.5 s in the EELS maps. The EELS maps were extracted by summing over an energy window of 0.2 eV. The EELS maps were processed with a multivariate weighted principal component analysis (PCA) routine.[1] Gaussian fitting was applied to EEL spectra in order to define the peak positions and full-width at half-maximums (FWHMs) of plasmon resonances. A JEOL ARM200F STEM/TEM with a post-specimen spherical aberration (Cs) corrector (CEOS GmbH) was also used to perform EFTEM thickness mapping using an energy-slit width of 10 eV and an exposure time of 2.44 s.

Electromagnetic simulations. A Matlab toolbox (MNPBEM) was used for the boundary element method simulations of EEL spectra.[2] The dimensions of the structures were set to the dimensions

observed in the experimental data. The bowtie structures in the simulations were constructed using two equal triangular nanoprisms with an edge length of 105 nm and a height of 20 nm. For the simplicity of complex simulations, an effective-medium approach was employed in BEM simulations. An electron-beam excitation with the beam energy of 200 keV was used in the simulations. BEM simulations of EEL spectra were performed in the energy range of 0.3–4 eV using 300 mesh. The structures including graphene membranes were simulated via the finite-difference time-domain (FDTD) method based on the exact solution of Maxwell's equations. A commercial software (Lumerical Inc.) was used for FDTD simulations performed using an x-polarized plane-wave light source. While the dielectric function of Al was estimated by the Drude-Lorentz model, the dielectric function of ML graphene was taken from Nelson *et. al.*.[3]

LC circuit model. As described earlier by Duan *et al*,[4] the junction or gap area of a bowtie is modeled as an LC tank oscillator with a parallel-connected nanoinductor (L_b) and nanocapacitor (C_g) in the LC circuit model, while both nanoprisms are modeled as LC tank oscillator circuits comprising of parallel-connected L_0 and C_0 (see Supplementary Figure S1). When two nanoprisms are separated with a gap, L_b is considered to be infinite. To simplify the LC model, the following assumptions are applied: (i) $L_b = k_1/w$, where k_1 is a fitting parameter involving the kinetic inductivity, length, and height of the junction. (ii) The geometric inductance is disregarded, since the kinetic inductance dominates in the narrow junctions. (iii) The gap is modeled as a parallel-plate capacitor expressed as $C_g= k_2/d$, where d is the width of the gap and k_2 is a fitting parameter involving permittivity and average cross-sectional area of the opposing faces of nanoprisms. (iv) If the nanoprisms are connected with a conductive junction, C_g is assumed to be constant.

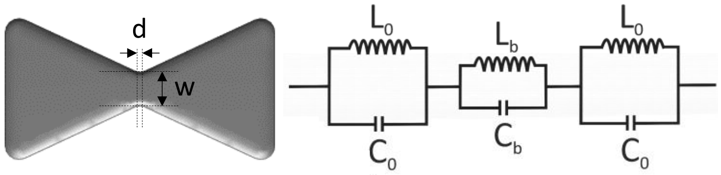

Supplementary Figure S1. A bowtie with a conductive junction and its corresponding LC circuit. Here, d and w represent the length and width of the junction.

The impedance (Z) of the LC circuit model shown above is obtained using the following equation.

$$Z = \left(i\omega(2L_0 + L_b)\left[1 - \omega^2\left(\frac{L_0L_b}{zL_0+L_b}\right)\left(2C_g + C_0\right)\right]\right)/((1 - \omega^2 L_0C_0)(1 - \omega^2 L_bC_b))$$

Here, several resonances of the bowties can be calculated by following the descriptions below:

For $\omega = \frac{1}{\sqrt{L_0C_0}}$, $Z = \infty$. In this case, there is not a net current flow from the tank oscillator, but the currents oscillate between the capacitor and inductor. This resonance condition corresponds to the dipolar dark mode and the dark λ resonance of bowties with and without a gap.

For $\omega = \frac{1}{\sqrt{L_bC_b}}$, $Z = \infty$. This resonance fulfills the condition for a charge transfer mode excited at connected nanoprisms. For the bowties with a gap, the charge transfer resonance does not occur, because $\omega = 0$ when $L_b = \infty$. The charge transfer mode is obtained by fitting the data to $\omega = \frac{1}{\sqrt{\left(\frac{k_1}{w}+a\right)C_g}}$, where k_1, a and C_g are fitting parameters.

For $\omega = \frac{1}{\sqrt{\frac{L_0L_b}{2L_0+L_b}(C_0+2C_b)}}$, $Z = 0$. At this resonance condition fulfilling the 3λ/2 mode, the combined impedance of the nanoprisms is aborted by the impedance of the load. The 3λ/2 resonance condition is simply described as $\omega = c\sqrt{bw + 1}$, where c and b are fitting parameters. The same condition

also enables obtaining dipolar bright mode if $L_b = \infty$. At this circumstance $\omega = \frac{1}{\sqrt{L_0(C_0+2C_g)}}$ for the dipolar bright mode.

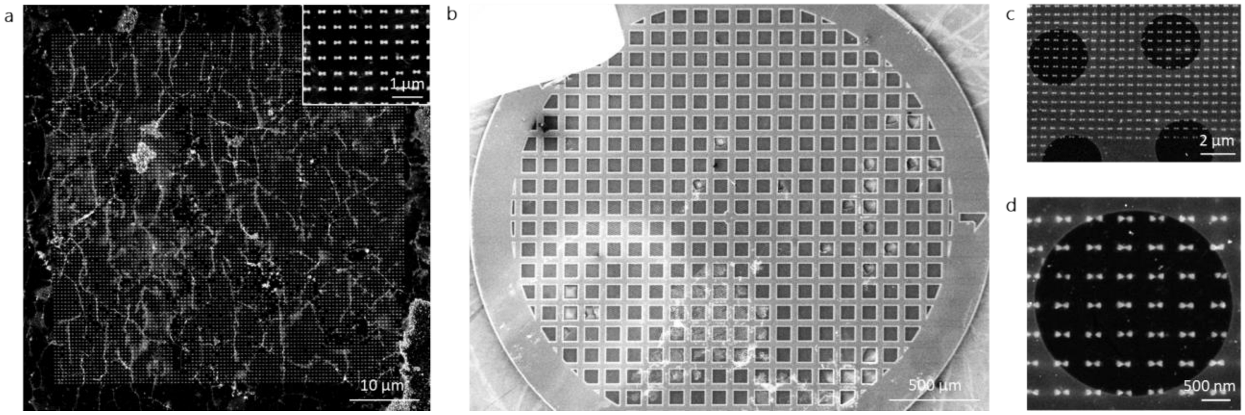

Supplementary Figure S2. (a) SEM image of a 50 µm×50 µm bowtie array on monolayer graphene CVD-grown on Cu foil. The inset shows the close-up SEM image of the bowties. (b) SEM image of the TEM grid that bowtie arrays on graphene were transferred. (c,d) HAADF images showing the bowties on suspended graphene attached to the quantifoil of the TEM grid.

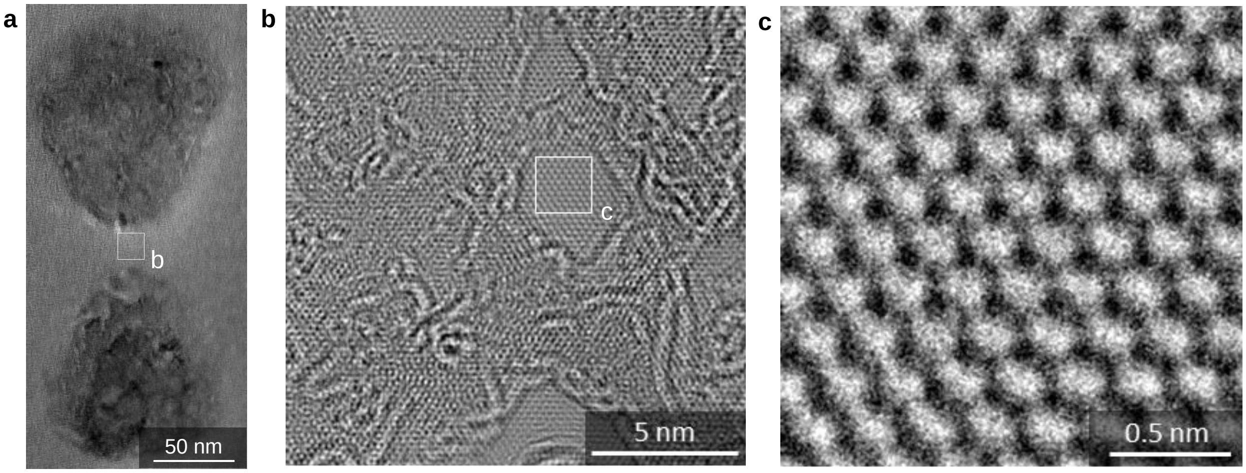

Supplementary Figure S3. (a) TEM image of an Al bowtie. (b,c) HRTEM images of monolayer graphene recorded at the areas marked in white squares.

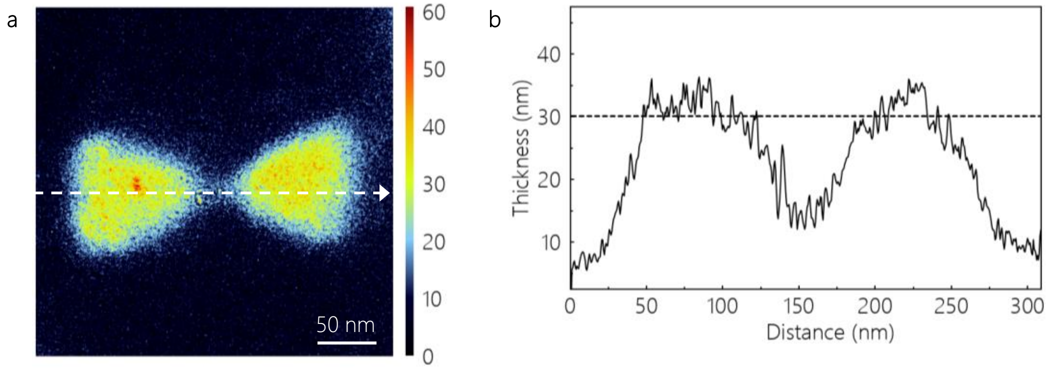

Supplementary Figure S4. (a) EFTEM thickness map for an Al bowtie. (b) Line profile along the white dashed line in panel a.

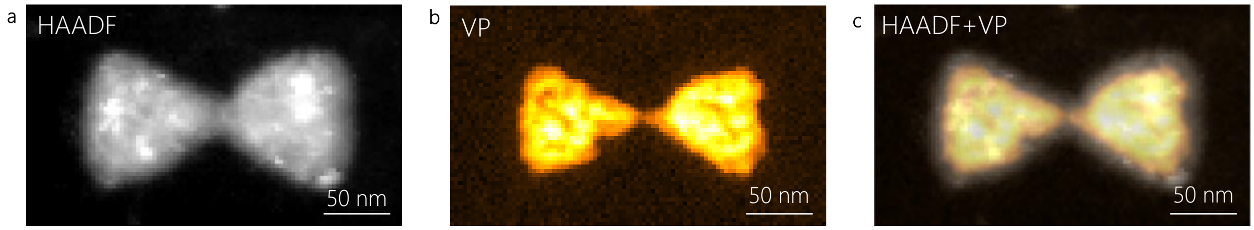

Supplementary Figure S5. (a) HAADF image of a bowtie with a junction. (b) Volume plasmon map of the bowtie in panel a. (c) Superimposed HAADF image and volume plasmon map.

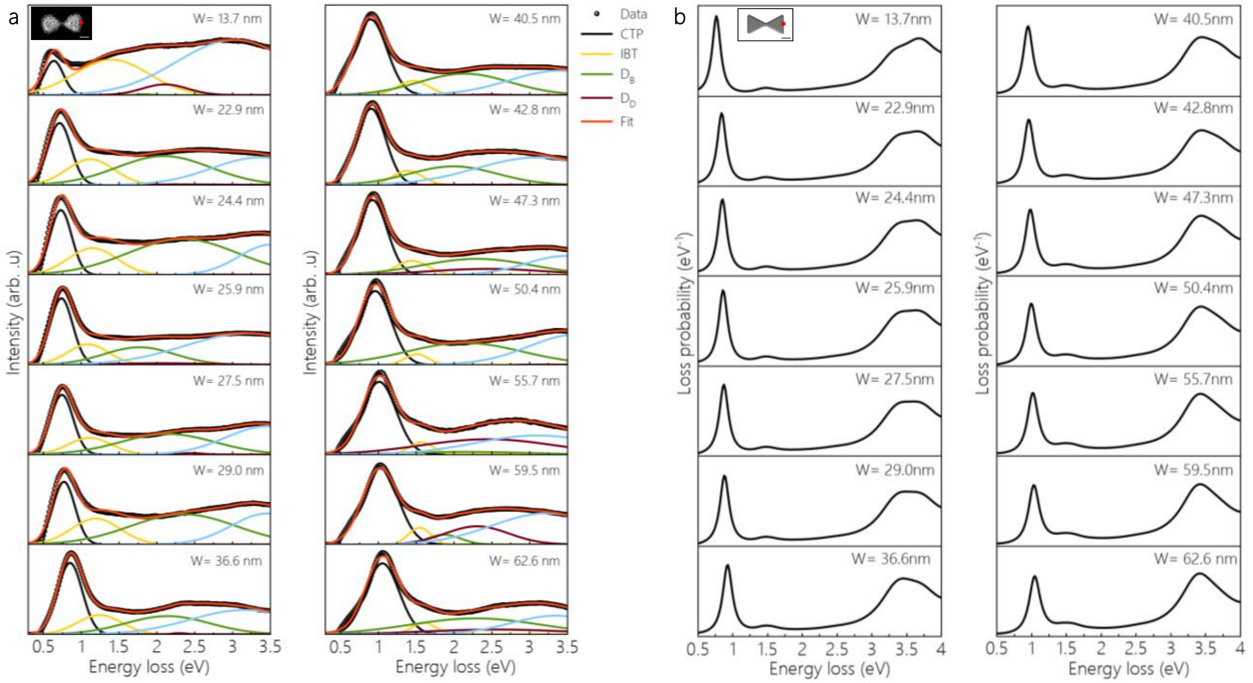

Supplementary Figure S6. (a) EEL spectra acquired at the edge of Al bowties (see inset) with different junction widths. (b) Simulated EEL spectra for Al bowties with similar sizes and junction widths.

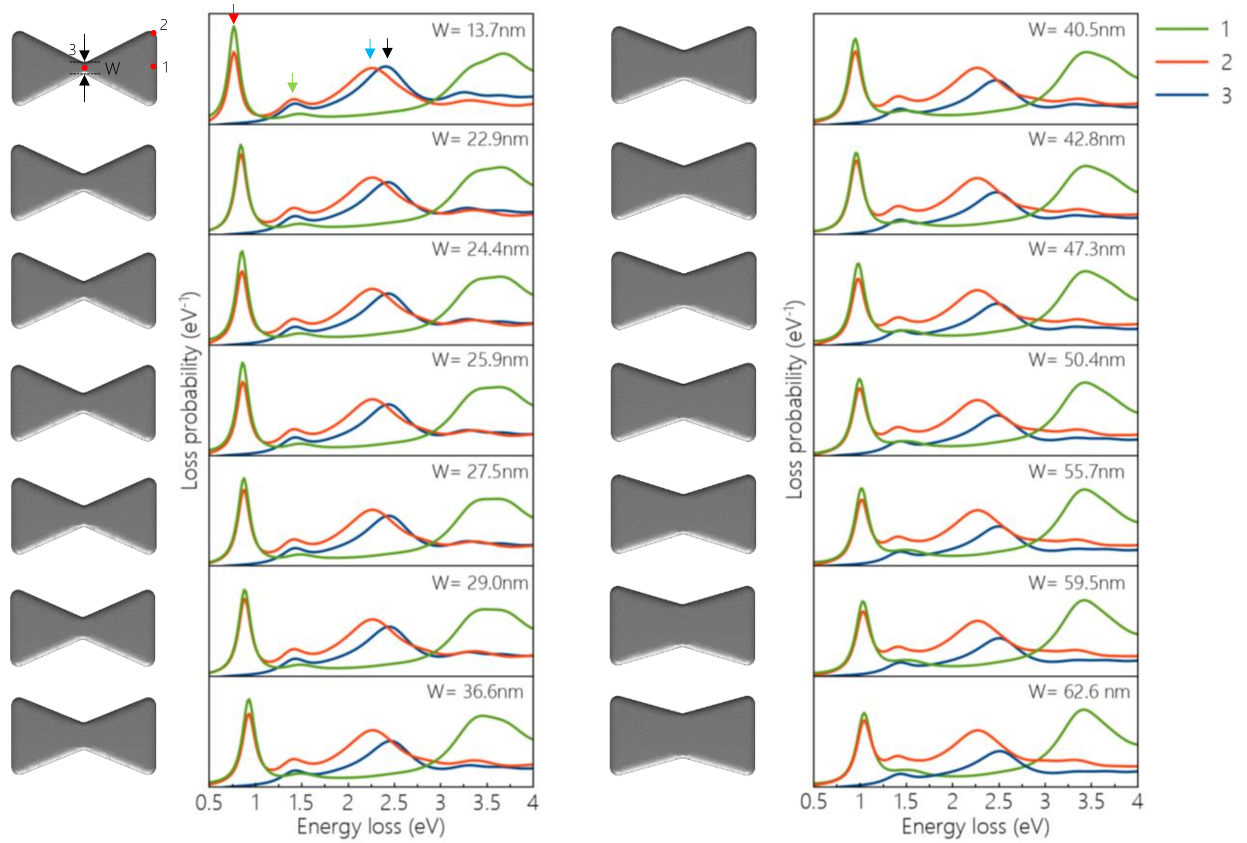

Supplementary Figure S7. Simulated EEL spectra for Al bowties with different junction widths. The red, blue and black arrows indicate the λ/2, 3λ/2 and λ resonances while the green arrow shows the interband transition of Al.

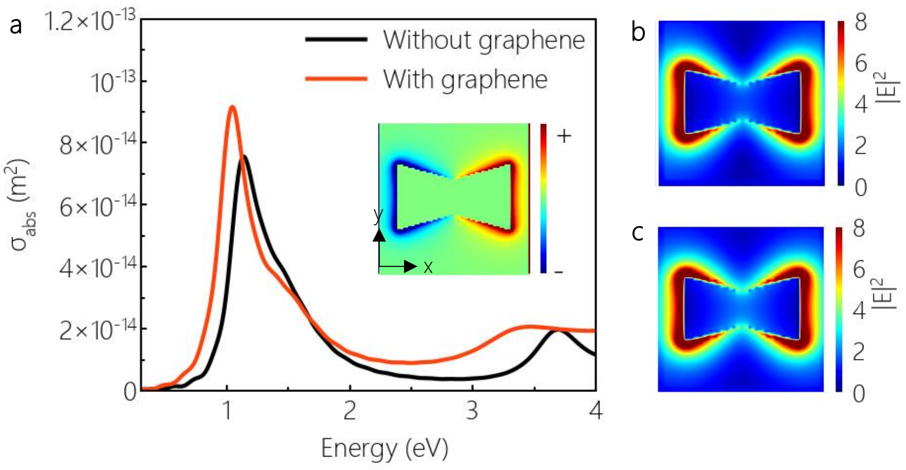

Supplementary Figure S8. (a) Absorption cross-section for a bowtie with and without monolayer graphene membrane. Inset shows the simulated surface charge distribution obtained at 1.04 eV for the bowtie on graphene. (b,c) Electric field maps calculated at the energies of 1.13 eV and 1.04 eV for bowties without and with graphene membrane, respectively.

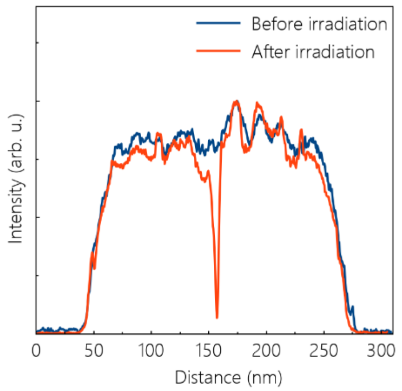

Supplementary Figure S9. Line profile recorded along the white dashed lines on the HAADF image in Figure 4 in the manuscript.

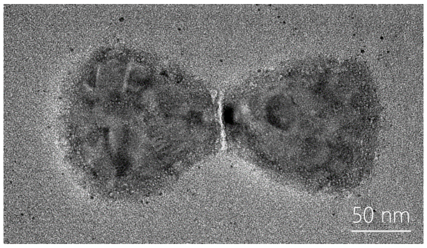

Supplementary Figure S10. TEM image of the bowtie after creating a gap by a focused electron beam.

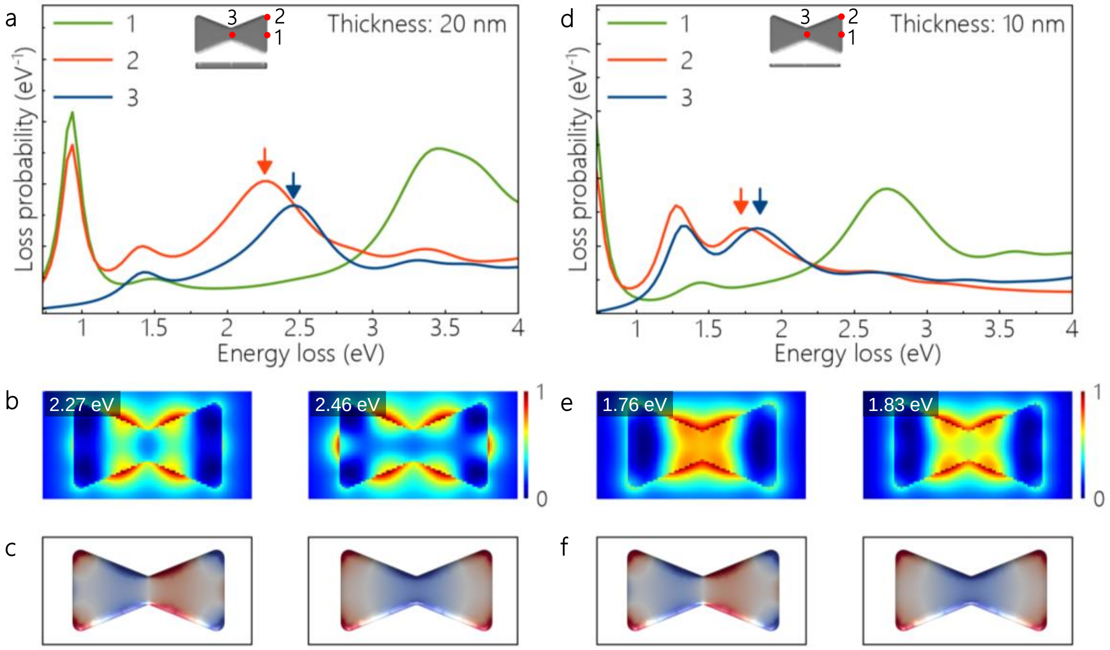

Supplementary Figure S11. (a) Simulated EEL spectra obtained at the positions marked on the model shown in the inset. Here, the thickness of connected nanoprisms is 20 nm. (b,c) Simulated EELS maps and eigenmodes corresponding to the 3λ/2 and λ resonances (see red and blue arrows on the EEL spectra) excited at the structure shown in panel a. (d) Simulated EEL spectra obtained at the positions marked on the model shown in the inset. Here, the thickness of connected nanoprisms is 10 nm. (e,f) Simulated EELS maps and eigenmodes corresponding to the 3λ/2 and λ resonances (see red and blue arrows on the EEL spectra) excited at the structure shown in panel d.

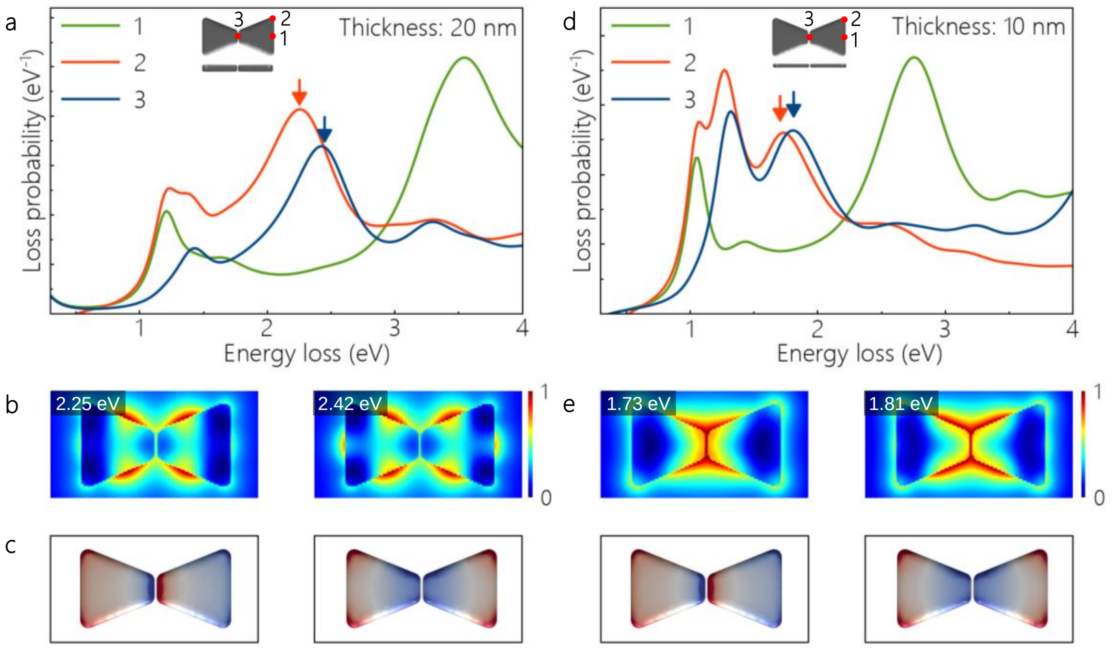

Supplementary Figure S12. (a) Simulated EEL spectra obtained at the positions marked on the model shown in the inset. Here, the thickness of disconnected connected nanoprisms is 20 nm. (b,c) Simulated EELS maps and eigenmodes corresponding to the dipolar bright and dipolar dark modes (see red and blue arrows on the EEL spectra) excited at the structure shown in panel a. (d) Simulated EEL spectra obtained at the positions marked on the model shown in the inset. Here, the thickness of disconnected nanoprisms is 10 nm. (e,f) Simulated EELS maps and eigenmodes corresponding to the dipolar bright and dipolar dark modes (see red and blue arrows on the EEL spectra) excited at the structure shown in panel d.

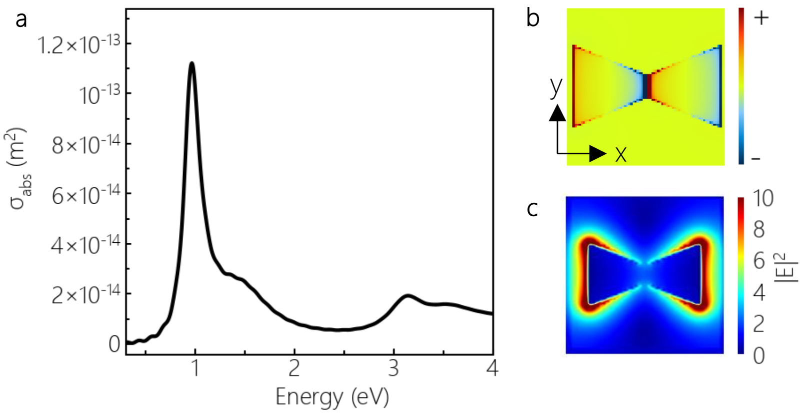

Supplementary Figure S13. (a) Absorption cross-section for an Al bowtie with a 3.4 nm gap on graphene. (b,c) Surface-charge distribution and electric-field map calculated at 0.97 eV.

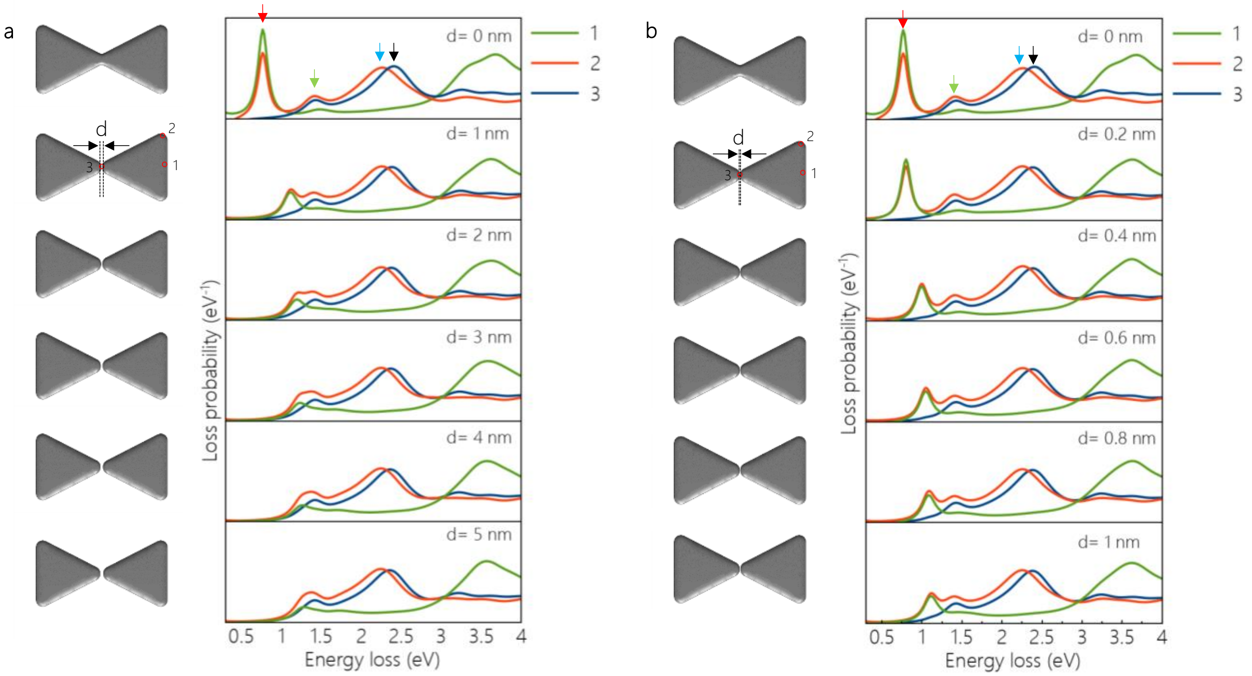

Supplementary Figure S14. (a) Simulated EEL spectra for Al bowties with different gap sizes varying from 0 nm to 5 nm. (b) Simulated EEL spectra for Al bowties with different gap sizes varying from 0 nm to 5 nm. The red, blue and black arrows indicate the λ/2, 3λ/2 and λ resonances, while the green arrow shows the interband transition of Al.

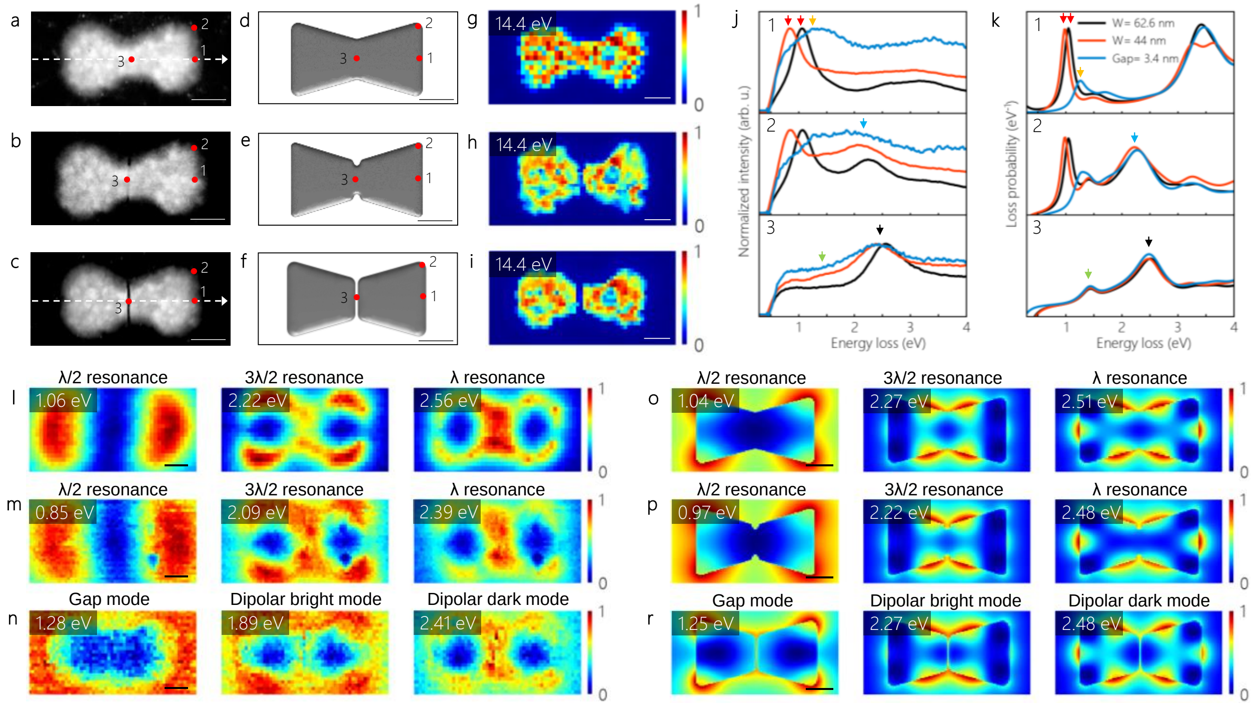

Supplementary Figure S15. (a) HAADF image of a bowtie with a junction width of 62.6 nm. (b) HAADF image of the same bowtie when the junction width is reduced to 40 nm. (c) HAADF image of the bowtie after creating a 3.4 nm gap. (d-f) Models corresponding to the structures in panels a-c. (g-i) Volume plasmon maps for structures shown in panels a-c, respectively. (j,k) Experimental and simulated EEL spectra extracted at positions marked with red dots in panels a-f, respectively. The red and orange arrows mark the CTP and gap modes. The blue arrows point to the 3λ/2 and dipolar bright modes, while the black arrows point to the λ and dipolar dark modes. The green arrow shows the IBT. (l-n) Experimental EELS maps created at different energies for the structures in panels a-c. (o-r) Simulated EELS maps calculated at different energies for the structures in panels d-f. The scale bars are 50 nm.

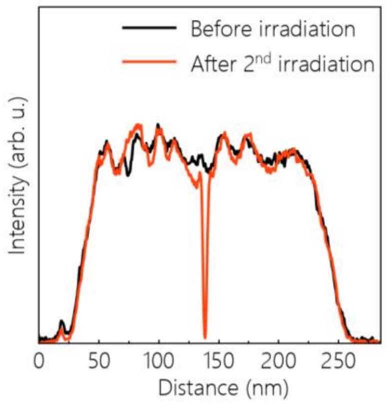

Supplementary Figure S16. Line profile recorded along the white dashed lines on HAADF image in Figure 5 in the manuscript.

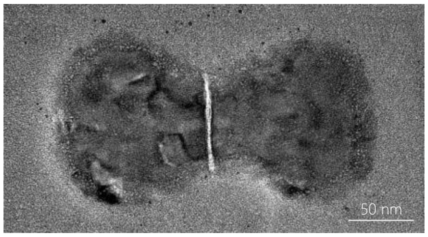

Supplementary Figure S17. TEM image of the bowtie after creating a gap by a focused electron beam (see Supplementary Figure S16c).

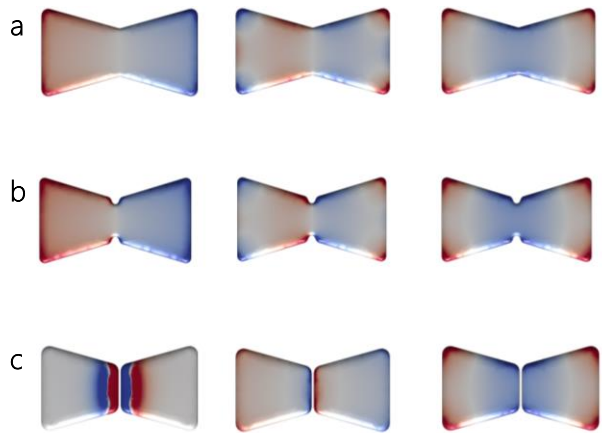

Supplementary Figure S18. Computed eigen modes corresponding the plasmon resonances shown in Supplementary Figure S15o-r.

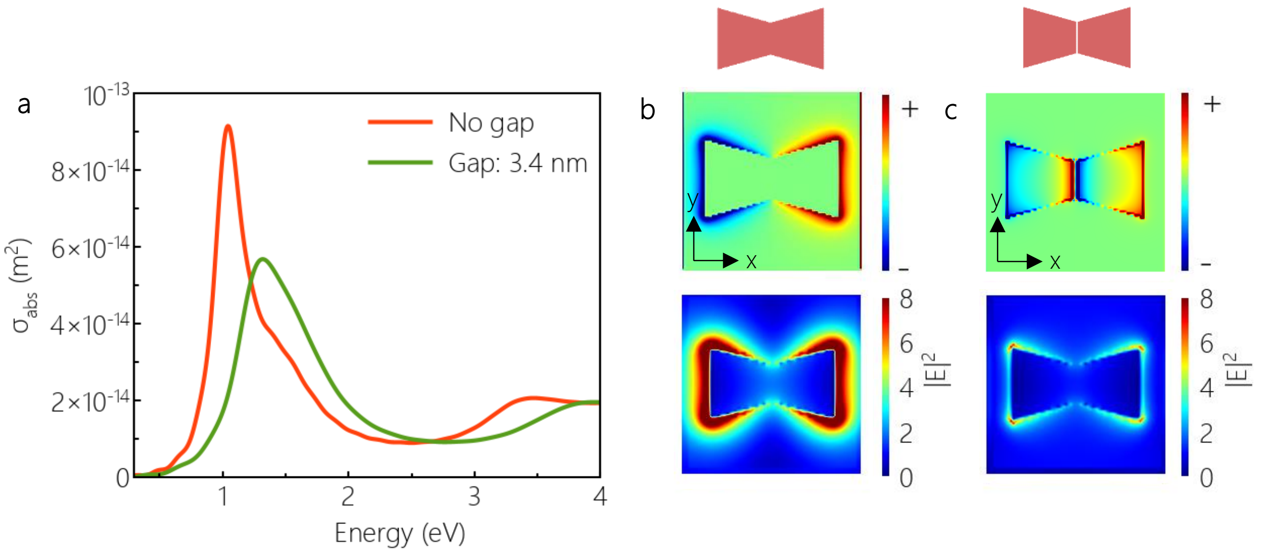

Supplementary Figure S19. (a) Absorption cross-sections for bowties without a gap and with a 3.4 nm gap on graphene. (b-c) Surface-charge distributions and electric-field maps calculated at 1.04 eV and 1.32 eV for bowties without a gap and with a 3.4 nm on graphene.

REFERENCES

1. Bosman, M.; Watanabe, M.; Alexander, D. T. L.; Keast, V. J., Mapping chemical and bonding information using multivariate analysis of electron energy-loss spectrum images. *Ultramicroscopy* **2006,** *106* (11), 1024-1032.

2. Hohenester, U., Simulating electron energy loss spectroscopy with the MNPBEM toolbox. *Computer Physics Communications* **2014,** *185* (3), 1177-1187.

3. Nelson, F. J.; Idrobo, J.-C.; Fite, J. D.; Mišković, Z. L.; Pennycook, S. J.; Pantelides, S. T.; Lee, J. U.; Diebold, A. C., Electronic Excitations in Graphene in the 1–50 eV Range: The π and π + σ Peaks Are Not Plasmons. *Nano Letters* **2014,** *14* (7), 3827-3831.
